# Rethinking the ASCO Resource-Stratified Cervical Cancer Screening Guidelines in the Context of Existing Health Infrastructure in Basic Settings

**DOI:** 10.1200/JGO.17.00025

**Published:** 2017-07-17

**Authors:** Megan Swanson, Peter Gimei, Megan Huchko

**Affiliations:** **Megan Swanson**, University of California at San Francisco, San Francisco, CA; **Peter Gimei**, Uganda Cancer Institute, Kampala, Uganda; and **Megan Huchko**, Duke University and Duke Global Health Institute, Durham, NC

## TO THE EDITOR:

*Journal of Global Oncology* has recently published resource-stratified clinical practice guidelines for secondary prevention of cervical cancer.^1^ The guidelines provide expert recommendations for cervical screening programs in basic, limited, enhanced, and maximal-resource settings.

In line with the WHO’s recommendation, the new ASCO guidelines for basic settings recommend human papillomavirus (HPV) testing, where feasible, as the primary screening modality given its simple interpretation and high sensitivity for cervical intraepithelial neoplasia and effectiveness at preventing progression to invasive cancer.^1-3^ The guidelines further stipulate that if HPV testing is not available or feasible, visual inspection with acetic acid (VIA) is an acceptable alternative.

Although ASCO should be commended for these streamlined, evidence-based, resource-stratified guidelines, we challenge two recommendations that we believe overlook opportunities to use existing health infrastructure in low- and middle-income countries. First, ASCO recommends VIA scale-up in settings where HPV testing is considered not feasible as a necessary step to create infrastructure for future HPV testing. We disagree with this recommendation. Given the increasing availability of feasible, acceptable HPV DNA tests that can be self-collected by women outside a clinic,^4,5^ we suggest that resources may be better spent by developing community-based HPV testing for primary screening, rather than scaling up widespread VIA. Cost-effectiveness modeling has demonstrated that HPV testing can be more cost-effective than VIA in Uganda.^6^

Many countries with basic resources available for cervical cancer screening have decentralized health care infrastructure, including lay health workers who provide basic health information and services. In Uganda, for example, the community-level providers, the Village Health Teams, have successfully assisted with education and HPV test provision in research settings. We posit that an HPV-based screening strategy can be designed to fit into the existing decentralized infrastructure, whereas scaling up VIA as primary screening would require training primary providers, equipping village health facilities, and overcoming cultural barriers to implement pelvic exam–based screening. Community-based self-administered HPV tests eliminate the need for skilled providers and equipment and allow programs to focus on the essential step of linking women with positive HPV tests to further pelvic exam–based evaluation (whether a triage test is used, women would at least need an exam to assess candidacy for treatment) and ablative or excisional treatment.

Second, ASCO recommends deferring the screening of pregnant women until they are postpartum, which misses a key opportunity to interact with an at-risk population. The ASCO guidelines advise waiting until 6 weeks postpartum, given the particular challenges of screening in pregnancy. There is a theoretical concern that women may have increased HPV prevalence during pregnancy secondary to immune changes.^7^ Thus, screening with HPV tests during pregnancy may lead to overtreatment. More challenging is the follow-up for positive tests because cryoablation and loop electrosurgical excision procedure are not performed in pregnancy.

However, the drawbacks of HPV testing in pregnancy must be weighed against the reality that pregnancy is often the only time women in low-income countries access medical care. According to United Nations Children’s Fund, 92% of pregnant Ugandan women, for example, have at least one prenatal visit with a skilled provider.^8^ However, in Uganda, only a fraction (15% to 40%) of women return for a routine 6-week postnatal visit.^9,10^ Because participation in prenatal care is nearly ubiquitous, failing to screen pregnant women is a missed opportunity. Of course, ensuring postpartum follow-up with a skilled provider for evaluation and ablative or excisional treatment of dysplasia is essential.

Although we applaud the creation of resource-stratified guidelines, we would argue that, especially for basic settings where creation of a new infrastructure to implement population-level screening is daunting, beginning by first considering innovative ways to use existing infrastructure is essential. In the case of Uganda, we posit that community health workers and self-administered HPV tests could potentially be used in creating a national community-based screening program. Another way to maximize existing health care use would be to add cervical cancer screening to standard, nearly universally attended prenatal care. Optimizing use of existing infrastructure will be essential for effective national screening programs, especially in basic settings with competing health priorities.
